# Minimal important improvement thresholds for the six-minute walk test in a knee arthroplasty cohort: triangulation of anchor- and distribution-based methods

**DOI:** 10.1186/s12891-016-1249-7

**Published:** 2016-09-13

**Authors:** J. M. Naylor, K. Mills, M. Buhagiar, R. Fortunato, R. Wright

**Affiliations:** 1 Whitlam Orthopaedic Research Centre, Orthopaedic Department, Liverpool Hospital, Locked Bag 7103, Liverpool BC, 1871 Sydney, NSW Australia; 2 South West Sydney Clinical School, UNSW, Sydney, Australia; 3 Ingham Institute of Applied Medical Health Research, Sydney, Australia; 4 Faculty of Medicine and Health Sciences, Macquarie University, Sydney, Australia; 5 Braeside Hospital, Hammondcare Group, Sydney, Australia; 6 Physiotherapy Department, Campbelltown Hospital, Sydney, Australia; 7 Occupational Therapy Department, Fairfield Hospital, Sydney, Australia

**Keywords:** Arthroplasty, knee, Arthroplasty, Six-minute walk test, Mobility test, Clinimetric

## Abstract

**Background:**

The 6-minute walk test (6MWT) is a commonly used metric for measuring change in mobility after knee arthroplasty, however, what is considered an improvement after surgery has not been defined. The determination of important change in an outcome assessment tool is controversial and may require more than one approach. This study, nested within a combined randomised and observational trial, aimed to define a minimal important improvement threshold for the 6MWT in a knee arthroplasty cohort through a triangulation of methods including patient-perceived anchor-based thresholds and distribution-based thresholds.

**Methods:**

Individuals with osteoarthritis performed a 6MWT pre-arthroplasty then at 10 and 26 weeks post-surgery. Each rated their perceived improvement in mobility post-surgery on a 7-point transition scale anchored from “*much better”* to *“much worse”.* Based on these responses the cohort was dichotomised into ‘improved’ and ‘not improved’. The thresholds for patient-perceived improvements were then identified using two receiver operating curve methods producing sensitivity and specificity indices. Distribution-based change thresholds were determined using two methods utilising effect size (ES). Agreement between the anchor- and distribution-based methods was assessed using kappa.

**Results:**

One hundred fifty-eight from 166 participants in the randomised cohort and 222 from 243 in the combined randomised and observational cohort were included at 10 and 26 weeks, respectively. The *slightly or more* patient-perceived improvement threshold at 26 weeks (an absolute improvement of 26 m) was the only one to demonstrate sensitivity and specificity results both better than chance. At 10- and 26-weeks, the ES based on the mean change score divided by the baseline standard deviation (SD), was an absolute change of 24.5 and 37.9 m, respectively. The threshold based on a moderate ES (a 0.5 SD of the baseline score) was a change of 55.0 and 55.4 m at 10- and 26-weeks, respectively. The level of agreement between the 26-week anchor-based and distribution-based minimal absolute changes was very good (k = 0.88 (95 % CI 0.81 0.95)).

**Conclusion:**

A valid threshold of improvement for the 6MWT can only be proposed for changes identified from baseline to 26 weeks post-surgery. The level of agreement between anchor- and distribution-based methods indicates that a true minimal or more threshold of meaningful improvement following surgery is likely within the ranges proposed by the triangulation of all four methods, that is, 26 to 55 m.

## Background

The 6-min walk test (6MWT) is a simple, objectively measured, physical test that is used to evaluate improvement in functional ambulation after TKA [1–8]. Simply stated, it is a test conducted in- or out-side on level ground where the participant is required to walk laps of a 25 or 30 m track [9]. Participants and observers are given standardised instructions on how to perform the test, and the distance walked over the 6-min period independent of rest periods is recorded. The use of the test in the TKA population arguably has content (face) validity as improvement in mobility is regarded as a primary goal of surgery [10] and rehabilitation after TKA surgery [11]. Further, construct validity for the test (that is, that the test is actually a measure of functional ambulation) for this population is derived from evidence that performance in the 6MWT has been shown to be an excellent predictor of performance in a more arduous 30-min walk test [11]. The test-retest reproducibility of the 6MWT is also excellent in TKA recipients [12] as well as in people with osteoarthritis awaiting arthroplasty [8, 13], and the test is highly responsive [8], indicating the test has the ability to detect change [14].

Interestingly though, despite demonstrating sound clinimetric properties and despite its common use both in the clinic [13, 15, 16] and in clinical trials [1–8], there are no published data on what may be minimal, moderate or large improvements in this test as perceived by the patient following TKA. Knowledge of what are considered small or large changes by the patient may be relevant for determining whether or not a change in therapy is indicated (at the level of the individual) as well as for sample size calculations for clinical trials [17–19]. Data exist on what minimal important differences (MID) are detectable for this test in this population using distribution-based methods based on observed scores [8, 20]. These methods express change in terms of a standardised metric such as 0.5 of a standard deviation (0.5SD) or the standardised error of measurement (SEM) [14, 21]. The SEM has been reported to be 28.5 m in people with knee osteoarthritis awaiting TKA [13]. Similar values have been reported six (25.5 m [12]) and eight weeks (26 m, [8]) post-TKA. Mizner et al [20] report the ES to be 0.66 (81 m) 1 year after surgery. However, distributional methods are criticised for ignoring the clinical importance of the magnitude of the change, for not including a measure of change as perceived by the patient, and for not necessarily being a ‘minimal’ change [14, 20]. An alternative method for determining MIDs, which does incorporate the views of the patient, is an anchor-based method. Anchor-based methods use an external reference (or anchor) by which to categorise respondents [14, 21]. Often these are patient-based and require the patient to qualify their global perception of change on a transition scale. Criticisms of anchor-based methods, however, are that they are prone to recall bias – that is, faulty recollection by the respondent [14, 21, 22] - and response-shift – a change in the respondent’s understanding of the construct being examined over time [22].

In light of the limitations of the methods to determine minimal or even moderate or large change thresholds, the use of multiple methods and triangulation of methodologies have been recommended [14, 21]. This study aimed, therefore, to define an improvement threshold for the 6MWT in a TKA cohort through a triangulation of methods using patient-perceived anchor-based improvement thresholds as well as distribution-based improvement thresholds.

## Methods

### Study design and setting

This study was nested within a multicentre, two-armed randomised controlled trial (HIHO) with a third non-randomised, observational arm [2] (http://clinicaltrials.gov ref NCT01583153). The controlled trial was designed to test the superiority of 10 days of inpatient rehabilitation together with a monitored home program on measured mobility over a monitored home program (usual care) alone following TKA. Those in the observational cohort received the same home program after their TKA. All participants provided informed, written consent and the study was approved by the human research ethics committees of the institutions involved. The protocol for the clinical trial is described in detail elsewhere [2]; a summary of the study procedures is provided herein.

### Participant screening and recruitment

Potential participants were screened by research personnel during their pre-admission visit approximately 4 weeks prior to surgery. Adults presenting to either of two metropolitan hospitals for a primary, unilateral TKA, with a primary diagnosis of knee osteoarthritis were eligible to participate in the RCT. People who were eligible, but declined to be included in the randomised arms of the study, were invited to participate in the observational arm whereby they received usual care. Socio-demographic and anthropometric data were obtained at this time. People who were unable to comprehend the study protocol, unable to perform exercises in an unsupervised environment, unable to attend one of three physiotherapy departments involved in the study, or who had a predisposition to be discharged to a rehabilitation facility (for example, they lived alone), were excluded from the study.

### Outcomes and testing procedures

After consent was obtained, each participant completed patient-reported surveys relevant to the larger study and completed a 6MWT on an outside 30 m straight track according to recommended testing procedures [9]. A practise 6MWT was not undertaken as all patients presenting for TKA at the study hospitals were required to perform the test at several time points whilst awaiting surgery as part of a waitlist management program [13, 15, 16]. At 10 weeks (randomised participants only) and 26 weeks (all participants) post-surgery, the 6MWT was repeated. Prior to testing, participants were asked to rate their perceived improvement in their mobility three ways; at 10 weeks, anchored to pre-surgery, then at 26 weeks, anchored to both pre-surgery and 10 weeks.

For rating patient global impression of improvement, we used an anchor-based method commonly recommended for determining the minimal important improvement [18, 23–25]. Participants were asked to rate their perceived improvement in mobility on a 7-point Likert scale. Each denoted whether they were ‘much worse’, ‘moderately worse’, ‘slightly worse’, ‘no change/same’, ‘slightly better’, ‘moderately better’, ‘much better’, compared to how they were prior to surgery. The global style of questioning used – ‘How does your walking compare to before surgery?’– was consistent with previous studies which have identified minimum thresholds for improvement for the 6MWT in other clinical populations [18, 19, 24].

### Preliminary analyses

Prior to analyses of the improvement thresholds, growth curve analyses were conducted to determine whether there were differences in the magnitude (model 1) and rate of change (model 2) in the 6MWT over the follow-up periods [26, 27]. These analyses allowed us to robustly deal with the change in sample size across different time points, but also indicated whether any improvement thresholds identified could apply across all time periods. The latter was important as MIDs are thought to be time-specific [14, 28, 29]. A third model was fitted to determine the influence of readily measurable patient variables on baseline 6MWT distance and/or the magnitude and rate of change over time (body mass index (BMI), age, gender, comorbidity count, baseline disease severity). This analysis was necessary as it would identify whether an improvement threshold could apply regardless of participant characteristics. For the purposes of this predictor model, rehabilitation group allocation was ignored as it was found to not significantly interact with baseline 6MWT or improvement in distance over time. To ensure best fit of the data, all models were fitted using an unstructured covariance structure, which requires no assumption in error structure [26].

### Analyses of thresholds

Anchor- and distribution-based approaches were utilised for determining meaningfulness of the improvement thresholds.

For the anchor-based method, identification of the thresholds and determining their acceptability were performed over three stages. Firstly, correlation between the absolute change scores from baseline to each follow-up period and the Likert scale was assessed using Spearman’s rank correlation. This was repeated for the relative change scores, where 6MWT distance was expressed as a percentage of baseline. While the optimal correlation coefficient for a typical MID analysis is conventionally regarded as >0.3 [14], due to the exploratory nature of this study, we chose to investigate the improvement thresholds that had any statistically significant (*p* <0.05) correlation.

Secondly, the improvement thresholds were investigated by dichotomising all participants into *“improved”* and *“not-improved”* groups. For the minimal group, the dichotomy was set with those reporting slightly improved or more (that is they reported slight, moderate or much better improvement) as the *improved group* and those reporting no change or worse as the *not-improved group*. The moderate group split occurred at the moderately better or more level, and the much better difference group only included those reporting they were much better. A priori, we had planned to identify the slight, moderate or much better thresholds in non-overlapping (independent) groups, however, too few people reported to be slightly better or even moderately better. Any conclusive analysis using these original categorisations was precluded, therefore, because such a small sample in the ‘slightly better’ group threatened the precision of the estimates obtained [30].

Thirdly, the 6MWT data, now dichotomised into those who had reported improvement or not, were plotted on a receiver operating characteristic (ROC) curve, with the improved group as the reference group on all occasions. This was done for all three improvement threshold groups. The area under the curve (AUC) and 95 % confidence intervals (CI) were calculated for each ROC curve in order to provide insight into the discriminatory power of the transition question. These were compared using DeLong’s statistic (D) to determine if using the slight, moderate or much better change was a more appropriate method for determining what would be useful clinically or scientifically. An AUC of 75 % or more has previously been proposed to be clinically useful [31]. The threshold of difference was then set using two methods: the first, the top left hand corner of the graph that results in the optimal combination of sensitivity and specificity, known as Youden’s method [32]; the second, the 80 % specificity method [33], selects the threshold that has a minimum of 80 % specificity while obtaining the highest possible sensitivity. Confidences intervals (CIs) for the sensitivity and specificity of each threshold were calculated using 500 bootstrap samples. Values greater than 50 indicated that the thresholds were better at identifying individuals who would (sensitivity) and would not (specificity) improve to a patient-perceived amount. ROC curves were calculated for the change in 6MWT both in absolute terms and as a percentage of the patient’s baseline value.

The distribution-based approach utilised the ES. There are two methods to this approach. The first examines the mean differences between pre- and post-surgical 6MWT distances and divides them by the standard deviation (SD) of the pre-surgery distance [34]. The second method is to determine 50 % of the SD of the baseline score, which correlates to a moderate effect [35]. This is a commonly used method to obtain a MID [36] and is based on a systematic review of 29 investigations across several disease conditions, which reported that the ES converged on 0.5 SD [35]. These methods were applied to both absolute and relative scores at 10- and 26-weeks post TKA. To examine the concordance in classifications between the anchor- and distribution-based MID thresholds, we used the kappa index of agreement [37]. To obtain 95 % confidence intervals for the kappas, we used 500 bootstrap samples.

## Results

Of the 243 participants included in the larger study, 166 and 77 belonged to the RCT and observational arms, respectively; 158 were available at the 10-week assessment (RCT participants only) and 222 were available at the 26-week assessment (RCT and observational combined). Table 1 summarises the characteristics of the cohort according to their study grouping (RCT or observational).

**Table 1:** Cohort characteristics

	Randomised cohort *n* = 166	Observational cohort *n* = 77	Entire cohort *n* = 243
Age, yrs, mean (sd)	67.0 (8.3)	66.7 (8.7)	66.9 (8.4)
Gender, female, n (%)	112 (68)	35 (45)	147 (61)
Body mass index, mean (sd)	34.7 (7.0)	33.0 (7.2)	34.2 (7.1)
Baseline 6-min walk test, mean (sd)	319.1 (109.1)	329.5 (114.0)	322.4 (110.6)
Baseline Oxford Knee Score	17.0 (7.1)	17.6 (7.7)	17.2 (7.3)
Comorbidity, yes, n (%)	127 (77)	53 (69)	180 (75)
Cardiovascular	116 (70)	47 (61)	163 (67)
Gastrointestinal	53 (32)	11 (14)	64 (26)
Other lower limb or back	43 (26)	21 (27)	64 (26)
Diabetes Mellitus	38 (23)	18 (23)	56 (23)
Respiratory	21 (13)	10 (13)	31 (13)

Key: Five most common comorbidities shown

### Growth curve analyses

The unadjusted mean preoperative distance was 322.4 (sd 110.6) m (Table 1) and the unadjusted distances achieved at 10 and 26 weeks were 375.5 (108.26) and 386.7 (113.2) m, respectively. The rates of improvement in the 6MWT changed significantly over time (refer to Appendix 1: Table 4). From 0 to 10 weeks, the adjusted mean increase in distance was 5.3 m per week for an average male. This rate slowed to a rate of 0.8 m improvement per week from weeks 10 to 26. While age and gender influenced preoperative 6MWT distance, they had no effect on the magnitude or rate of change. These results indicated that any significant thresholds that were identified would apply regardless of differences in the participant characteristics included in the model, but owing to the effect of time on improvement, any proposed threshold would be time-specific.

### Anchor-based estimation of improvement thresholds

#### Correlation of the transition scale with measured change

While the global transition scale was significantly correlated with the absolute and relative changes in 6MWT distance from baseline to 10- and 26-weeks, the correlation coefficients were small (Table 2). Further, there was no correlation between the changes in 6MWT from 10-weeks to 26-weeks and the transition scale. As such, the determination of improvement thresholds in the period between the 10- and 26-week follow-ups was excluded from further analysis.

**Table 2:** Spearman’s correlation coefficient between the transition scale, and absolute and relative change in 6MWT distance

Change in 6MWT	Rating on transition scale		
	Baseline to 10-weeks	Baseline to 26-weeks	10-weeks to 26-weeks
Absolute Baseline to 10-weeks	0.297 (*p* < 0.001)	NA	NA
	*n* = 156		
Relative Baseline to 10-weeks	0.259 (*p* < 0.001)	NA	NA
	*n* = 156		
Absolute Baseline to 26-weeks	NA	0.259 (*p* < 0.001)	NA
		*n* = 211	
Relative Baseline to 26-weeks	NA	0.239 (*p* < 0.001)	NA
		*n* = 211	
Absolute10-weeks to 26-weeks	NA	NA	0.03 (*p* = 0.73)
			*n* = 139
Relative 10-weeks to 26-weeks	NA	NA	0.054 (*p* = 0.526)
			*n* = 139

*NA* not applicable

#### Categorisation of improved versus not improved participants

**Fig. 1:**
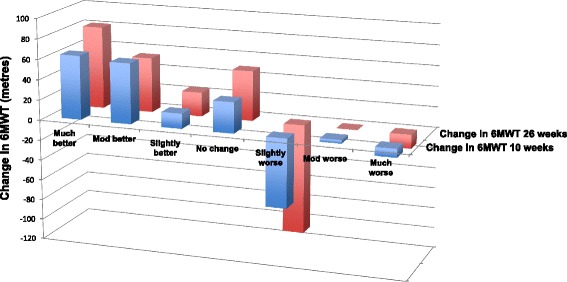
Mean change in 6MWT distance for each category of the transition scale for baseline to 10-weeks post op (foreground) and baseline to 26-weeks (distance). Sample size varied greatly for each category. At 10-weeks, much better *n* = 85, moderately better *n* = 43, slightly better *n* = 12, no change *n* = 6, slightly worse *n* = 2, moderately worse *n* = 7 and much worse *n* = 2. At 26-weeks, much better *n* = 143, moderately better *n* = 36, slightly better *n* = 9, no change *n* = 9, slightly worse *n* = 4, moderately worse *n* = 3, much worse *n* = 7

At the 10-week assessment, there were 140 (89 %), 128 (81 %) and 85 (54 %) people included in the slightly or more, moderate or more and much better improvement categories, respectively. At 26 weeks, there were 188 (85 %), 179 (81 %) and 143 (64 %) in each of the threshold categories. Figure 1 indicates the mean deterioration or improvement in 6MWT distance observed between categories is not linear; in other words, there is not a graduated increase or decrease from category to category. Through reference to the wide range of maximum negative and positive change observed within each category (Table 3), it can be seen that some people in the ‘slightly improved or more’ category (that is, they reported they were *slightly better* or *more*), demonstrated greater improvement or deterioration than people in the ‘much better’ category (that is, those who reported they were *much better*).

**Table 3:** Area under the curve for ROC curves as well as slight or more, moderate or more and much better important difference for both the absolute and relative changes for the 6MWT

Important change	Max negative and positive changes in distance^a^	AUC (95 % CI)	Youden’s method	Sensitivity (95 % CI)^b^	Specificity (95 % CI)^b^	80 % Specificity method	Sensitivity (95 % CI)^b^	Specificity (95 % CI)^b^
10 weeks Absolute								
Slight or more (*n* = 140)	−148.0 m to 290.0 m	70.1 % (55.58, 84.7)	11.5 m	70.2 (62.41, 78.39)	66.7 (40, 86.67)	53.0 m	48.9 (41.13, 57.45)	80.0 (60, 100)
Moderate or more (*n* = 128)	−148.0 m to 290.0 m	72.3 % (51.15, 83.35)	11.5 m	74.4 (66.67, 82.95)	70.4 (51.85, 85.19)	53.0 m	51.9 (42.64, 60.47)	81.4 (66.67, 92.59)
Much better (*n* = 85)	−148.0 m to 252.0 m	62.8 % (53.85, 71.78)	11.5 m	79.1 (70.93, 87.21)	48.6 (36.39, 60)	106.5 m	29.1 (19.16, 38.37)	80.0 (71.43, 88.57)
10 weeks Relative								
Slight or more (*n* = 140)	−92.6 to 486.0 %	67.3 % (51.21, 83.44)	5.1 %	66.7 (58.16, 74.13)	66.7 (40, 86.67)	15.1 %	49.7 (40.43, 58.16)	80.0 (60, 100)
Moderate or more (*n* = 128)	−92.6 to 486.0 %	70.7 % (59.05, 82.32)	5.1 %	70.5 (62.02, 78.29)	70.4 (51.85, 85.19)	15.5 %	48.8 (40.31, 56.59)	81.5 (66.67, 94.54)
Much better (*n* = 85)	−92.6 to 486.0 %	60.7 % (51.53, 69.9)	5.7 %	54.3 (44.29, 65.71)	70.5 (63.95, 83.17)	49.1 %	23.3 (15.12, 32.56)	80.0 (70.68, 88.57)
26 weeks Absolute								
Slight or more (*n* = 188)	−242.3 m to 370.0 m	72.4 % (62.05, 82.7)	26 m	72.9 (66.21, 79.26)	65.2 (45.54, 82.61)	64.5 m	50.0 (43.09, 56.91)	82.6 (67.28, 95.65)
Moderate or more (*n* = 179)	−242.3 m to 370.0 m	70.6 % (60.33, 80.94)	6.5 m	81.6 (75.98, 87.15)	56.3 (38.98, 71.88)	71.0 m	48.0 (41.05, 55.31)	81.3 (67.28, 95.65)
Much better (*n* = 143)	−194.0 m to 370.0 m	64.7 % (56.59, 72.77)	8.5 m	84.4 (78.01, 90.07)	41.4 (31.43, 54.29)	110.5 m	35.5 (27.66, 43.63)	81.4 (72.86, 90)
26 weeks Relative								
Slight or more (*n* = 188)	−93 to 515.2 %	71.7 % (60.64, 82.79)	11.3 %	65.4 (58.51, 72.09)	73.9 (56.52, 89.24)	18.3 %	52.1 (45.74, 59.04)	82.6 (67.28, 95.65)
Moderate or more (*n* = 179)	−93.2 to 515.2 %	69.6 % (58.74, 80.4)	1.5 %	82.1 (76.54, 87.71)	56.3 (37.5, 71.88)	27.9 %	40.78 (33.78, 48.6)	81.3 (65.62, 93.75)
Much better (*n* = 143)	−91.3 to 515.2 %	63.4 % (55.07, 71.78)	1.54 %	85.1 (79.06, 90.78)	41.4 (30, 52.86)	61.0 %	21.3 (14.18, 27.66)	84.3 (74.29, 84.29)

^a^Change in distance from pre-operative 6MWT. Negative integers and percentages <100 % indicate 6MWT is less than preoperative values. Positive integers and percentages >100 % indicate farther distance

^b^Specificity or Sensitivity values ≤50.0 indicate that the important improvement threshold is no better than chance at classifying individuals as improved or not

#### Area under the curve, specificity and sensitivity analyses

**Fig. 2:**
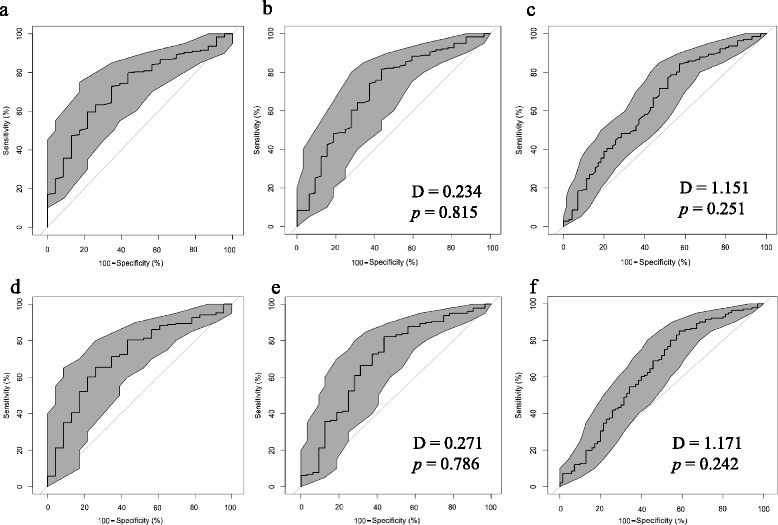
ROC curves depicting Absolute (*top row*) and Relative (*bottom row*) improvement in 6MWT distance (*black line*) and 95 % CI of specificity (*grey shading*) at 26-weeks post-operative. Panels **a** and **d** depict the slight or more important difference **b** and **e** depict moderate or more important and **c** and **f** depict much better important difference. DeLong’s test compares AUC of graph to that of the minimal important difference

The AUCs indicated that the improvement thresholds were not highly discriminatory with respect to measured changes in the 6MWT (60–75 %) regardless of whether relative or absolute change was used (Table 3). Further, there was no difference in discriminatory power between slight or more, moderate or more and much better definitions of improvement (Fig. 2). The Youden’s and 80 % specificity method resulted in different thresholds of slight or more, moderate or more and much better important change. Slight improvement or more at 26-weeks was the only set of thresholds where the specificity and sensitivity were both greater than 50 % for absolute and relative change in both the Youden and 80 % specificity methods. That is, they were the only thresholds considered to have a sensitivity and specificity which were uniformly better than chance, regardless of the analysis method. The absolute values indicated that a “slight improvement or more” ranged from 26 to 64.5 m improvement in distance, or a relative increase between 11.3 and 18.3 %. For the remaining 10- and 26-week thresholds either the sensitivity or specificity were poor suggesting that they are sub-optimal for identifying clinically useful improvement in a cohort regardless of the improvement category used (Table 3).

### Distribution-based estimation of improvement thresholds

At 10-weeks, the ES based on the mean change score (52 m) divided by the baseline SD (110 m) was 0.5. This equivocated to an improvement of 24.5 m or 12.7 % being considered an important change. At 26-weeks this method (64.8 m/110.8) resulted in an ES of 0.6 and proposed threshold of 37.9 m or 19.6 % change. The distribution MID based on 0.5SD of baseline scores was an improvement of 55.0 m or 14.6 % of baseline scores at 10-weeks post-surgery and 55.4 m or 14.3 % at 26-weeks post-surgery.

### Agreement between anchor- and distribution-based methods

The kappa level of agreement between the 26-week anchor- and distribution-based minimal change ranged from moderate to strong for absolute change. Agreement between the 80 % specificity ROC method and ES distribution approach exhibited the lowest agreement (k = 0.67 (95 % CI 0.57, 0.76)) and the highest agreement occurred between the Youden ROC method and ES distribution approach (k = 0.88 (0.81, 0.95)). Similarly, when thresholds of relative change were examined, agreement between anchor- and distribution-based approaches ranged from moderate to almost perfect. The lowest agreement was between the Youden ROC method and ES distribution approach (k = 0.69 (0.6, 0.78)) and highest was between the Youden ROC method and 0.5SD distribution approach (k = 0.91 (0.85, 0.96)).

## Discussion

To our knowledge, this is the first study to attempt to explore the possibility that patient-perceived improvement thresholds exist for the 6MWT in a TKA cohort. Specifically, we have explored improvement and change thresholds for the 6MWT, using multiple analytical approaches and at two clinically relevant time periods: 10-weeks post-surgery, a time when formalised rehabilitation is typically concluding, and at 26 weeks post-surgery, a time when recovery is typically plateauing [3, 6, 7].

Further, our cohort characteristics signify an elderly population of people with end-stage osteoarthritis with significant impairment as indicated by the very low mean baseline Oxford scores (mean 17 from a maximum of 48), and the poor baseline walk tests which are well below the typical distances (582 m) measured in healthy 70-year olds [11]. These characteristics, including their comorbidities, typify TKA populations captured locally [3, 4] as well as those captured internationally [6–8]. Our observations, therefore, should be both useful to clinicians involved in the rehabilitation of TKA recipients and be broadly generalizable.

By using both anchor-based and distribution-based approaches and then assessing the level of agreement between the thresholds obtained by each approach, we have identified a *slight or more improvement* threshold at 26-weeks post-surgery for the 6MWT in a TKA cohort. Based on triangulation of all four methods (two ROC approaches utilising patient-perceived change, and two distributional approaches), and considering only the anchor-based and distributional thresholds with good agreement, it appears that the true threshold of a minimally important change is between 26 m and 55 m. Interestingly, and probably importantly, the threshold range we have identified appears consistent with patient-perceived change thresholds for the 6MWT determined in other patient populations using anchor-based methodologies. For patients with heart disease, it has been estimated to be 25 m [18]. In older adults with mobility impairments, a small meaningful change has been found to be 19 to 22 m and a more substantial change has been found to be 47 to 49 m [19]. An MID of 25 m was identified for patients with COPD [24].

In determining the contribution this study makes to this area, our study has strengths and limitations. The strengths of our study lie in the comparatively large sample size, its prospective, longitudinal design, and the inclusion of participants from both arms of our combined randomised and observational study - the latter enhancing the generalisability of our findings. We also used multiple methods to establish the one threshold we did identify whilst considering the potential confounders of time and patient characteristics. Further, our study describes an improvement threshold in the 6MWT post-TKA that can be applied at the level of the individual. The use of the ROC curve approach allows the identification of important patient level-change, whereas approaches only applying distribution-based methodologies necessarily confine their changes to group-level change only [16, 36].

That we identified a range over which small improvement may be considered to have occurred as opposed to a single ‘cut-off’ figure is unusual when determining MIDs, but may be considered quite useful. This is because it allows flexibility in how we perceive improvement for the individual and within groups, acknowledging that there are multiple non-medical variables or life events that may influence a person’s recovery post-TKA. Thus, there is not likely to be a single MID threshold that is universally representative. We also note that it is likely that future researchers in this area (arthroplasty, 6MWT and MID) will confront the same issue we faced with too few people reporting slight improvement, thus necessitating *slight or more* improvement threshold-type categorisations. This is because for many if not all TKA cohorts, very large improvements in various outcomes, including mobility, are typically seen [4, 8, 20].

Another difficult challenge in this area is applying a global question which captures all elements of improvement. Whilst we applied a global anchor which would allow us to compare our findings to others exploring change thresholds for the 6MWT, it may not have captured all the elements of improvement (or deterioration) in walking ability as perceived by the patient (and this would be the case for previous studies applying a similar anchor). Consequently, a lack of ability of our global question to capture all elements of improvement may have contributed to the weak correlations observed between the transition responses and measured improvements in walk distance. The 6MWT is essentially a test of gait speed; improvement may have occurred in other dimensions such as movement quality and, thus, not have been detected by the 6MWT or, for that matter, any other of the time-based mobility tests such as the timed up-and-go or 15 m walk test commonly used to test mobility after TKA [38]. It would appear a more specific question around improvement in speed per se or the use of a mobility test that is not time-based may be required to secure a greater correlation between measured change and perceived change, and, thus, achieve greater precision in a patient-perceived improvement threshold. Of course recall bias or response shift may also have contributed to the weak correlations observed, and this is not likely to be helped by a different global question. It should also be acknowledged that it is known that there is even poor concurrent validity between performance measures and what patients perceive they can do after TKA [20], thus, a more precise patient-perceived anchor or improvement for the 6MWT may never be found.

## Conclusions

In conclusion, though the 6MWT is commonly used to evaluate recovery after TKA, uncertainty exists as to what is considered a minimal or even large improvement as perceived by the patient. Using multiple methods and subsequent triangulation of these methods, the likely minimum threshold about which patient-perceived improvement from pre-surgical status can be considered to have occurred is between 26 and 55 m at approximately six months after surgery.

## Appendix

**Table 4:** Growth curve analyses for the 6MWT

Parameter	Estimate	95 % CI	Sig
Intercept	490.0	398.2 to 581.8	<0.001
Magnitude of change	73.8	50.2 to 97.3	<0.001
Rate of change over time	−20.5	−32.0 to -9.0	0.001
Gender	62.4	39.0 to 85.8	<0.001
Age	−2.9	−4.2 to −1.5	<0.001

Post-operative average distance of 6MWT at 10-weeks post-surgery can be calculated using the following equations where males are coded “0” and females are “1”

Males: 490 + (1 × 73.8) + (1x -20.5) + (0 × 62.4) + (66.2 × −2.9), where 66.2 is the average age

Females: 490.0 + (1 × 73.8) + (1x -20.5) + (1 × 62.4) + (66.8x -2.9), where 66.8 is the average age

## Abbreviations

6MWT: 6-min walk test
AUC: Area under the curve
MID: Minimal important difference
ROC: Receiver operating characteristic
SEM: Standardised error or measurement
TKA: Total knee arthroplasty
